# Analysis of Glomerular IgG Subclasses Switch in Idiopathic Membranous Nephropathy Classified by Glomerular Phospholipase A2 Receptor Antigen and Serum Antibody

**DOI:** 10.1155/2021/9965343

**Published:** 2021-08-30

**Authors:** Hao-yuan Cui, Chao Li, Hang Li, Yu-bing Wen, Lin Duan, Yan Li, Xi-wei Yan, Yu-ting Hu, Li-meng Chen, Xue-mei Li

**Affiliations:** Nephrology Division, Peking Union Medical College Hospital, Beijing 100730, China

## Abstract

**Background:**

The role of IgG subclass in idiopathic membranous nephropathy (IMN) was unclarified. Recent study found IgG subtype switches from IgG1 to IgG4 in the early pathological stage in IMN. The profile of IgG subclass in phospholipase A2 receptor- (PLA2R-) related and PLA2R-unrelated IMN was unrevealed. Our study is aimed at testifying whether IgG subclass switch existed in PLA2R-related and PLA2R-unrelated IMN, respectively.

**Methods:**

Our study retrospectively enrolled 157 Chinese patients with biopsy-confirmed IMN between September 2017 and November 2019. We measured glomerular PLA2R antigen and serum anti-PLA2R antibody to classify the patients into PLA2R-related (*n* = 132) and PLA2R-unrelated (*n* = 25) subgroup. We evaluated glomerular IgG subclass by immunofluorescence (IF) predominance. Our study defined IgG subclass deposition as predominant if the IF score was higher than the other three and ≥1 +, or as codominant if the IF intensity was equal to any other and ≥1 +. We explored the relationship between IF predominance of glomerular IgG subtype and electron microscopic (EM) stages of IMN.

**Results:**

We did not find statistical difference of predominant or codominant rate (pre/co-rate) among EM stages in any subclass (*P* > 0.05). Pre/co-rate of IgG3 linearly associated with EM stage in total and PLA2R-related subgroup (*P* = 0.044, *P* = 0.013). PLA2R-related subgroup showed higher IgG4 intensity (2.1 ± 0.6 vs. 1.6 ± 0.7, *P* = 0.001) and pre/co-rate of IgG4 in stage 1 (97% vs. 57%, *P* = 0.015) than PLA2R-unrelated group. We found no difference of IgG subclass pre/co-rate in different EM stages or linear association between pre/co-rate of IgG1, IgG2, IgG4, and EM stages (*P* > 0.05).

**Conclusions:**

Pre/co-rate of IgG3 declined with EM stage in total and PLA2R-related subgroup. We did not find IgG subclass switches from IgG1 to IgG4 in either IMN patients or subgroups.

## 1. Introduction

Idiopathic membranous nephropathy (IMN) is a leading cause of nephrotic syndrome among the adult population [1, 2]. M-type phospholipase A2 receptor (PLA2R), the dominant autoantigen of IMN with mostly specific IgG4 autoantibodies, has been studied widely since discovered in 2009 [3]. The exact role of different IgG subclass in IMN remained elusive. In 2013, Huang et al. reviewed pathological data in 157 membranous nephropathy patients. They implied major IgG subtype deposited in glomerular changed from IgG1 to IgG4 during the process of IMN [4]. However, the IgG subclass switching was observed in some but not all studies. Hihara et al. suggested IgG1 might dominate in situ while anti-PLA2R antibody was not shown by analyzing a small group of PLA2R-related IMN [5]. In 2016, Kattah et al. revealed IgG4 predominate in 5 recurrent PLA2R-related IMN and declared no subclass switch [6]. Huang et al. failed to test circulating antibody or glomerular antigen of IMN. It was unclear whether the IgG subtype switching was present in subgroup stratified by different antigen.

In this study, we investigated the IgG subclass distribution of PLA2R-related and PLA2R-unrelated IMN in different EM stages. To verify the IgG subclass switching in IMN, we illustrate the profile of IgG subtype in IMN and further investigate the IgG subclass switching in PLA2R-related and PLA2R-unrelated IMN.

## 2. Materials and Methods

### 2.1. Study Population

**Figure 1:**
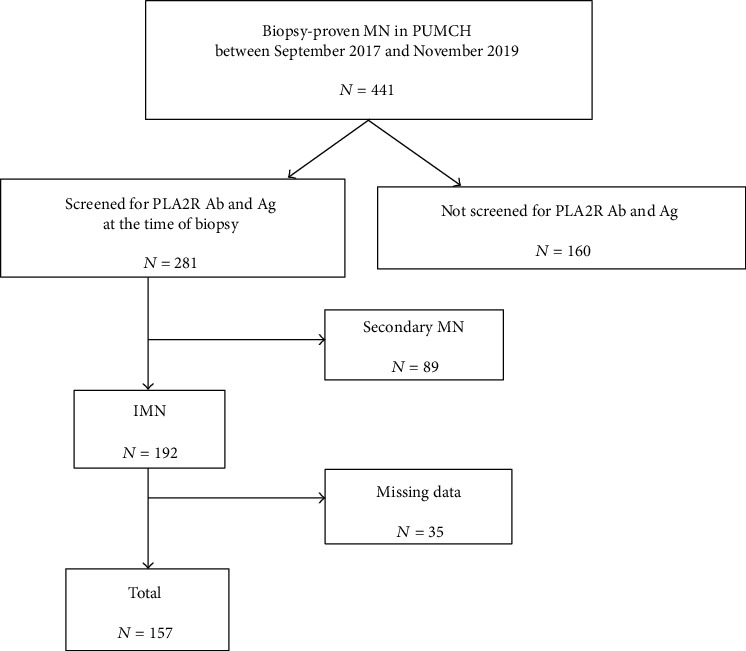
Flow diagram showing patients included in this study.

Our study retrospectively enrolled 157 patients with biopsy-proven IMN hospitalized in the Peking Union Medical College Hospital (PUMCH) between September 2017 and November 2019. The study was approved by the PUMCH ethical committees (No. S-K014). The inclusion criteria were as follows: diagnosis of IMN confirmed by renal biopsy; serum anti-PLA2R antibody was detected at the time of diagnosis. Patients with known secondary MN, such as hepatitis B/C virus infection, drug/toxin exposure, autoimmune disease, or malignancy, were excluded (Figure 1). We collected clinical data from medical records at the time of diagnosis, including age at biopsy, gender, serum creatinine (Scr), albumin (Alb), and 24h urine protein (24hUP).

### 2.2. Detection of Circulating Anti-PLA2R Ab

We collected the serum for anti-PLA2R antibody assay before treatment and performed with enzyme linked immunosorbent assay (ELISA) kit (EUROIMMUN, Lübeck, Germany) according to the manufacturer's instruction. We set up the cut-off value of the positive result as above 14 relative light units per milliliter (RU/mL).

### 2.3. Kidney Biopsy

We conducted standard kidney biopsy [7, 8]. Tissue for light microscopy was routinely stained with hematoxylin and eosin, Masson, Jones methenamine silver, and periodic acid-Schiff reagent. Specimen for immunofluorescence (IF) was cut at 5 *μ*m in freezing microtome. We performed direct IF with fluorescein isothiocyanate- (FITC-) conjugated antibodies to human IgG, IgA, IgM, C3, C4, C1q, *κ*-, and *λ*-light chains, fibrinogen, and albumin (Dako, Copenhagen, Denmark) for 30 minutes in 37°C. We used indirect IF to detect IgG subclass in situ. The slice was conducted with 1 : 50 mouse anti-human IgG1, IgG2, IgG3, and IgG4 (Invitrogen, Camarillo, CA) at 4°C overnight, followed by 1 : 25 FITC-conjugated rabbit anti-mouse IgG (Dako, Copenhagen, Denmark) at 37°C 2 h. Renal tissue for EM was performed according to the standard protocol [9].

We semiquantitatively scored IF staining from 0 to 3 + (0 negative, 1 + weak staining, 2 + moderate staining, and 3 + strong staining). The predominant deposition was defined if the score was higher for one IgG subclass than the other three and ≥1 +. IF staining was categorized as codominant if more than one IgG subclasses were equal in intensity and ≥1 +. The EM stage was classified according to the Ehrenreich and Churg classification [9]. We divided different EM stages into 4 groups: 1, 2, 3, and 4. Stage 1 included EM stages 1 and 1-2. Stage 2 included EM stages 2 and 2-3. EM stage 3-4 and stage 4 were classified into stage 4 for its immune complex was started to reabsorb.

### 2.4. Glomerular PLA2R Ag Expression

We processed PLA2R staining by indirect IF using mouse anti-human PLA2R Ab (Sigma, Gallen, Switzerland) at a dilution of 1 : 200 overnight at room temperature, followed by FITC-conjugated rabbit anti-mouse IgG (Dako, Copenhagen, Denmark) at a dilution of 1 : 25 at 37°C for 2 h.

### 2.5. Definition of PLA2R-Related and PLA2R-Unrelated IMN

PLA2R-related IMN was defined as positive circulating antibody to PLA2R by ELISA and/or glomerular PLA2R antigen by IF. PLA2R-unrelated IMN was defined if both serum antibody and glomerular antigen exposure were negative.

### 2.6. Statistical Analyses

Clinical parameters were expressed as medians and quartiles (P_25_, P_75_) for nonnormally distributed continuous variables. The Mann-Whitney *U* test was performed for comparison between two subgroups of continuous variables. Differences in categorical variables were analyzed using the Chi-squared test or Fisher's exact when appropriate. Trends were explored by linear-by-linear association test. All tests were two-sided and *P* values < 0.05 were considered statistically significant. Statistical analyses were performed by the SPSS statistical software package, version 20.0 (SPSS Inc., Chicago, IL).

## 3. Results

### 3.1. Clinical and Pathological Characteristics of Total IMN Patients

Among the 157 patients with IMN, 57% (*n* = 89) were male and 43% (*n* = 68) were female, with a median age of 51 (38, 58) years at the time of diagnosis. The medium Alb was 27 (23, 32) g/L, the medium 24hUP was 4.75 (2.21, 7.4) g/L, and the medium Scr was 72 (61, 86.5) *μ*mol/L. Among them, intensities of IgG1, IgG2, IgG3, and IgG4 were 1.4 ± 0.7, 0.5 ± 0.7, 1.0 ± 0.8, and 2.0 ± 0.6, respectively. Pre/codeposition of IgG subclass in different EM stage was shown in Table 1. The pre/co-rate of IgG3 linearly associated with the EM stage (*P* = 0.044).

### 3.2. Clinical and Pathological Data of PLA2R-Related and PLA2R-Unrelated Subgroup

#### 3.2.1. Serology and Histology of PLA2R

The clinical and pathological data of two subgroups were shown in Table 2. The positive rate of PLA2R was 56% (88/157) in circulation and was 76% (119/157) in situ (see Table 3). Patients were divided into PLA2R-related (*n* = 132, 84%) and PLA2R-unrelated (*n* = 25, 16%) subgroup. The differences showed no statistical significance of age, gender, Alb, 24hUP, and Scr of different EM stages between two subgroups (Table 2). The anti-PLA2R ab titers of different stages in serum-positive patients showed no statistical significance.

#### 3.2.2. Fluorescence Intensity of IgG Subclass Staining

**Figure 2:**
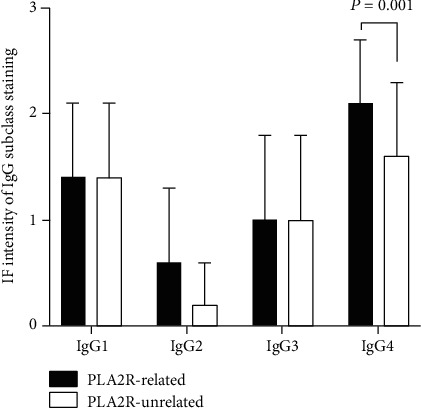
The fluorescence intensity of IgG subclasses compared in PLA2R-related and PLA2R-unrelated group.

**Figure 3:**
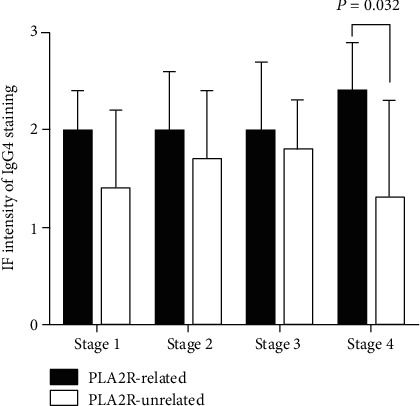
The fluorescence intensity of IgG4 compared in PLA2R-related and PLA2R-unrelated group.

IF staining of IgG subclass was illustrated in Figure 2. PLA2R-related subgroup showed higher glomerular deposition of IgG4 (2.1 ± 0.6 vs. 1.6 ± 0.7, *P* = 0.001) than PLA2R-unrelated subgroup. Among different EM stage, the intensity of the IgG4 deposit was much higher in stage 4 of PLA2R-related subgroup than that of PLA2R-unrelated subgroup (2.4 ± 0.5 vs. 1.3 ± 1.0, *P* = 0.032) (see Figure 3).

#### 3.2.3. IgG Predominant/Codominant Distribution and Trend Exploration

**Figure 4:**
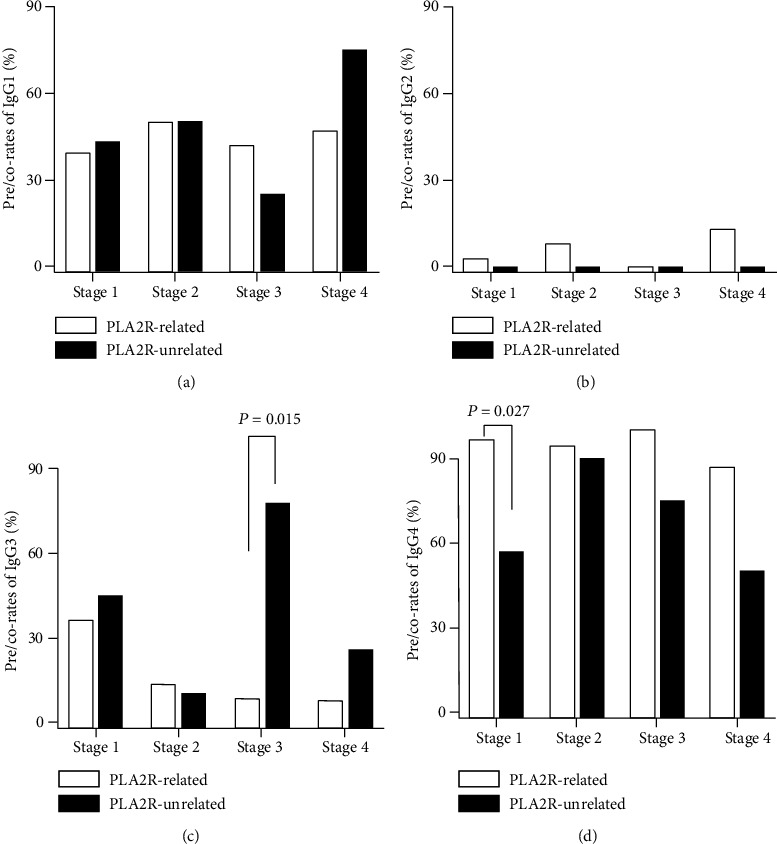
Predominant/codominant deposits of IgG subclasses in different EM stages in PLA2R-related and PLA2R-unrelated subgroup.

Pre/codeposition of IgG subclass in different EM stage was illustrated in Figure 4. In PLA2R-related subgroup, pre/co-rate of IgG4 was higher in stage 1 (97% vs. 57%, *P* = 0.015), and pre/co-rate of IgG3 was lower in stage 3 (8% vs. 75%, *P* = 0.027) than that in PLA2R-unrelated subgroup (see Figures 4(d) and 4(c)). The pre/co-rate of IgG3 showed a linear association with EM stage in PLA2R-related group (see Table 4).

## 4. Discussion

The main result of our study was IgG3 showed a declining trend from EM stages 1 to 4 in PLA2R-related subgroup. There were three possible explanations. Firstly, more evidences indicated that IgG3 played a key role in the early stage of immune diseases. Previous study delineated that human body produced a fixed order of IgG subclass in immune response, namely, IgG3 > IgG1 > IgG2 > IgG4 [10]. Elevated IgG3 in serum might predict the initial stage of an immune disease, such as acute rheumatic fever [11]. IMN incidence presents an increase trend [12] and is associated with high level of PM 2.5 exposure [13]. Researchers speculated environmental hazards acted B cell to produce specific antibody against PLA2R-expressing on both alveolar epithelial cell and podocyte [14]. In 2012, Debiec et al. reported an early recurrent PLA2R-related IMN caused by exclusively IgG3 [15]. Our data present a similar result in a homogeneous cohort and added another brick to the role of IgG3 in early stage of PLA2R-related IMN.

Secondly, our result fitted the established phenomenon of epitope spreading in PLA2R-related IMN [16]. The theory was described as cysteine-rich (CysR) exposed in the early stage of PLA2R-related IMN, then spread to C-type lectin domain 1(CTLD1) and C-type lectin domain 1 (CTLD7) to enhance the immune response. IgG1, 2, 3, 4 combined CysR and CTLD1 while IgG4 only acted on CTLD7 [16].

Thirdly, some researchers detected IgG1 and IgG3 subtype of PLA2R-related IMN but IgG4 were absent [17]. Although IgG4 was the predominant Ig of PLA2R-related IMN in situ, few evidences elucidated its pathogenicity. Thus, our result indicated that IgG3 might play a potent role in the early stage of PLA2R-related IMN.

We did not discover IgG subclass switch from IgG1 to IgG4 in IMN as reported by Huang et al. [4]. The pre/co-rate of IgG4 in EM stage 1 was obviously high in our study than that in Huang et al.'s study (89% vs. 55%). Three possible reasons might contribute to the discrepancy. Firstly, the different inclusion criteria could bring selection bias. The clinical data were not provided in Huang et al.'s work. We failed to analyze the difference of two study populations. Secondly, the discrepancy might come from the distribution of EM stages between two studies (*χ*^2^ = 24.85, *P* < 0.001). The difference mainly existed in early stages (*n* = 38, 24.2% vs. *n* = 58, 50.9% in stage 1) and middle stages (*n* = 100, 63.7% vs. *n* = 39, 34.2% in stages 2-3) between our data and Huang et al.' s. Thirdly, the definition of early EM stage (stage 1) was different. Huang et al. emphasized they separated the “true” stage 1 by redefining it as electronic dense absent between subepithelial. Our cohort might include more advanced patients of stage 1. However, the pre/co-rate of IgG4 was comparable between stages 1 and 2 (89% vs. 94%) in our study, even in PLA2R-related group that was higher of stage 1 than that of stage 2 (97% vs. 95%). It was unlikely that the discrepancy of EM stage definition gave rise to the gap on pre/co-rate of IgG4 in stage 1 between two studies. We assumed the discrepancy just reflected ethnic differences [18]. Huang et al. provided no serum or pathological profiles of PLA2R. To further explore IgG subtype switch of different antigens, we divided total study population into PLA2R-related and PLA2R-unrelated subgroup.

We found no IgG subclass switch from IgG1 to IgG4 in PLA2R-related or unrelated subgroup. In PLA2R-related subgroup, IgG4 was the predominant IgG subclass of all stages. Our results were identical to other Asia studies reported by Hayashi et al. [19] and Dong et al. [20]. For PLA2R-unrelated group, our data indicated that it was a heterogeneous subgroup. Several studies described potential autoantigens such as aldose reductase, SOD2, thrombospondin type 1 domain-containing 7A (THSD7A), exostosin1/exostosin2 (EXT1/2), and neural epidermal growth factor-like 1 protein (NELL-1) besides PLA2R [21–25]. The IgG subtype distributions of those antigens were not explicitly discussed except THSD7A [26]. In the early stage of EXT1/2 positive MN, the pathological and clinical manifestation might present as PLA2R-negative IMN until the secondary cause revealed [24]. Researchers recently discovered that anti-NELL-1 could also induce IMN but prevailing with IgG1. The pathological feature of PLA2R-unrelated subgroup should be investigated separately according to different autoantigens in future research.

The novelty of our study was that we distinguished PLA2R-related group from IMN and found out IgG subclasses distribution of PLA2R-related/PLA2R-unrelated IMN among different EM stages. The declined IgG3 deposition with EM stage in PLA2R-related subgroup might suggest a new perspective to understand the disease. The limitations of our study must be mentioned. Firstly, our profile of IgG subclasses might not well represent the population due to the limited sample size, especially stage 3-4. Secondly, we chose indirect IF to measure IgG subclasses on glomerular. The method was sensitive but might cause false positives. Thirdly, we did not screen other autoantibody of MN because of limited serum or tissue sample. It would be interesting to study glomerular IgG subclass of PLA2R-unrelated MN population by increasing the sample size.

## 5. Conclusions

IgG3 showed a declining trend in PLA2R-related IMN. We did not observe the glomerular IgG subclass switch from IgG1 to IgG4 in either total IMN patients or subgroups.

## Figures and Tables

**Table 1:** Predominance/codominance and EM stage in IMN.

IgG subclass	Pathological stage				*P* values	
	*n* (%) pre/co-rate				Chi-square test	Linear-by-linear association test
	I (*n* = 38)	II (*n* = 84)	III (*n* = 16)	IV (*n* = 19)		
IgG1	15 (40%)	42 (50%)	6 (38%)	10 (53%)	0.577	0.530
IgG2	1 (3%)	6 (7%)	0 (0%)	2 (11%)	0.452	0.450
IgG3	14 (37%)	15 (18%)	4 (25%)	2 (11%)	0.065	0.044
IgG4	34 (89%)	79 (94%)	15 (94%)	15 (79%)	0.186	0.281

**Table 2:** Comparison of clinical characteristics between two subgroups.

Characteristic	PLA2R-related (*n* = 132)	PLA2R-unrelated (*n* = 25)	*P* value
Age (yrs), M (P_25_, P_75_)	51 (37, 58)	49 (40.5, 57.5)	0.867
Men, *n* (%)	73 (55%)	16 (64%)	0.421
Alb (g/L), M (P_25_, P_75_)	28 (23, 32)	25 (20.5, 35)	0.378
24hUP (g/d), M (P_25_, P_75_)	4.7 (2.3, 7.3)	5.4 (1.8, 8.6)	0.878
Scr (*μ*mol/L), M (P_25_, P_75_)	73 (61, 87)	72 (63, 86)	0.859
*EM stage number, (%)*			0.386
1	31 (24%)	7 (28%)	
2	74 (56%)	10 (40%)	
3	12 (9%)	4 (16%)	
4	15 (11%)	4 (16%)	

**Table 3:** Prevalence of serum anti-PLA2R antibody and glomerular staining of PLA2R.

		Serum anti-PLA2R antibody		
		+	-	Total
PLA2R on glomerular	+	75	44	119
	-	13	25	38
Total		88	69	157

**Table 4:** Predominance/codominance and EM stage in PLA2R-related and PLA2R-unrelated subgroup.

IgG subclass	Pathological stage				*P* values	
	*n* (%) predominance or codominance				Chi-square test	Linear-by-linear association test
PLA2R-related	I (*n* = 31)	II (*n* = 74)	III (*n* = 12)	IV (*n* = 15)		
IgG1	12 (39%)	37 (50%)	5 (42%)	7 (47%)	0.767	0.768
IgG2	1 (3%)	6 (8%)	0 (0%)	2 (13%)	0.493	0.434
IgG3	11 (35%)	14 (13%)	1 (8%)	1 (7%)	0.087	0.013
IgG4	30 (97%)	70 (95%)	12 (100%)	13 (87%)	0.469	0.380
PLA2R-unrelated	I (*n* = 7)	II (*n* = 10)	III (*n* = 4)	IV (*n* = 4)		
IgG1	3 (43%)	5 (50%)	1 (25%)	3 (75%)	0.640	0.574
IgG2	0 (0%)	0 (0%)	0 (0%)	0 (0%)	NA	NA
IgG3	3 (43%)	1 (10%)	3 (75%)	1 (25%)	0.081	1.000
IgG4	4 (57%)	9 (90%)	3 (75%)	2 (50%)	0.331	0.835

NA: not available.

## Data Availability

Comprehensive data are presented in the manuscript, table, and figures. The datasets used and analysed during the current study are available from the corresponding author on reasonable request.
